# A Questionnaire Survey on the Prevalence and Parents’ Perceptions of Respiratory Allergies in a 3- to 16-Year-Old Population in Wuhan, China

**DOI:** 10.3390/jcm11164864

**Published:** 2022-08-19

**Authors:** Shuyan Guo, Yin Wang, Hao Chen, Nan Huang, Wenjing Li, Dongxia Ma, Yaqi Yang, Shuchen Zhang, Rongfei Zhu

**Affiliations:** 1 Department of Allergy, Tongji Hospital, Tongji Medical College, Huazhong University of Science and Technology, Wuhan 430030, China; 2 Institute of Allergy and Clinical Immunology, Tongji Hospital, Tongji Medical College, Huazhong University of Science and Technology, Wuhan 430030, China

**Keywords:** allergic rhinitis, asthma, prevalence, questionnaire, perception, Wuhan

## Abstract

(1) Background: The prevalence of allergic rhinitis (AR) and asthma has increased rapidly in China. However, perceptions of respiratory allergies and barriers to their management have not attracted enough attention. (2) Objective: To investigate the prevalence of, parents’ perceptions of and their unmet needs for information concerning respiratory allergies in a 3- to 16-year-old children population. (3) Methods: A cross-sectional survey was conducted from June to July 2021 in three schools in Wuhan, China. A total of 1963 participants were recruited through cluster sampling for their parents to complete an online questionnaire regarding respiratory allergic symptoms. The diagnosis of respiratory allergies was based on self-reported symptoms and face-to-face physician evaluation. All the participants with respiratory allergies were asked to complete the Brief Illness Perception Questionnaire (B-IPQ), the Asthma Knowledge Questionnaire (AKQ) and a questionnaire regarding their unmet needs for disease management. (4) Results: The prevalence of respiratory allergies was 29.3% (576/1963) in the 3- to 16-year-old population, among whom AR accounted for 25.7%; asthma, 1.8% and AR-complicated asthma (AR&Asthma), 1.9%. The total B-IPQ score was 40.2 ± 10.9 in the participants with respiratory allergies, and there were no differences among the AR, asthma and AR&Asthma groups (all *p* > 0.05). The B-IPQ score correlated significantly with symptom onset time and a history of atopic dermatitis (*p* < 0.01). Nearly one fifth, 18.9%, of the participants with respiratory allergies never went to hospital for treatment, but those with higher B-IPQ scores were more likely to seek professional treatment (*p* < 0.001). The accuracy rates of AKQ were 72.5% in the participants with asthma and 76.7% in those without asthma (*p* = 0.147). Among the 576 participants with respiratory allergies, 568 (98.6%) had tried to obtain disease-management information from online platforms, and 55.5% (315/568) were dissatisfied with current platforms; the reasons included incomprehensive contents of illness (45.7%), lack of voice from leading experts (40.3%), too many advertisements (37.5%) and similar contents on different platforms (36.8%). (5) Conclusions: The prevalence of respiratory allergies is high in the 3- to 16-years old population in Wuhan, China. Yet the parents’ perceptions of respiratory allergies and knowledge of asthma are insufficient. It is crucial to increase parents’ awareness of the illness and facilitate their access to truly informative and professional platforms.

## 1. Introduction

Respiratory allergies are common chronic diseases in children. Epidemiological studies have shown that the prevalence of respiratory allergies increased quickly in China in the past 20 years [1,2,3]. A telephone-based study in 11 major cities found that the self-reported prevalence of allergic rhinitis (AR) increased from 11.1% in 2005 to 17.6% in 2011 [1]. The prevalence of pediatric asthma also increased from 0.9% in 1990 [4] to 3.0% in 2010 [5], and it is estimated that an increase in the prevalence rate of childhood asthma of just 1% will translate into 3 million more childhood asthmatics because of the large childhood population [6]. However, the prevalence of respiratory allergies varies greatly in different regions of China. For example, the prevalence of AR was 16.8% in Guangzhou (southern China), while it was only 3.9% in Xi’an (northern China) [7]. Meanwhile, there are studies suggesting that children are more likely to be affected by respiratory allergies than adults [8,9]. A study conducted in 2009 showed that the questionnaire-based AR prevalence among 3- to 6-year-old children in Wuhan was 27.1%, and the prevalence of allergen prick test-confirmed AR children was 10.8% [10]. Respiratory allergies have become a big challenge to society; it is reasonable to predict that considerably more people are going to be affected.

Despite the high prevalence of respiratory allergies, these diseases have not received enough attention from the public in China. It is well-known that perceptions of chronic diseases are a driver of patient behavior and may influence treatment outcomes [11,12]. In most cases, respiratory allergies are not fatal diseases; only a small proportion of patients will go to hospital to seek professional medical advice. Consequently, the AR and asthma control rates are low in real-world studies [13]. Many studies have demonstrated that AR and asthma had significant negative impacts on individuals’ quality of life and could lead to severe financial burden to the society, especially when AR and asthma are not controlled [14,15,16,17].

To better manage respiratory allergies and minimize their impacts on individuals, it is crucial to draw patients’ attention to the diseases. Traditionally, physicians have raised awareness during physician–patient interactions that take place in hospital or clinical settings. Patients receive information on disease management passively from doctors. Now the internet has permeated every aspect of our daily lives. Naturally, most patients tend to seek online medical help from different platforms instead of or before going to hospital. Online services heighten the awareness of respiratory allergies. However, they also bring new challenges to both patients and doctors [18].

To date, there are limited data on patients’ perceptions of respiratory allergies in China. In this cross-sectional study, we investigated the prevalence of and their parents’ perceptions of respiratory allergies in 3- to 16-year-old children. We also investigated their unmet needs for online platforms.

## 2. Materials and Methods

### 2.1. Study Design

This was a cross-sectional questionnaire-based study conducted from June 2021 to July 2021 in Wuhan. Students from three schools were enrolled by cluster sampling. All the students and their parents were invited to participate in the study. The demographic data and symptoms of respiratory allergies were collected through an online questionnaire. In this questionnaire, there were 16 questions regarding respiratory allergic symptoms (7 questions related to AR and 9 related to asthma) [19]. If the children had suspicious symptoms of respiratory allergies [any yes answer to any of the 16 questions], they received further face-to-face evaluation by our physicians, and their parents were invited to finish the Brief Illness Perception Questionnaire (B-IPQ) and the Asthma Knowledge Questionnaire (AKQ). The parents also needed to finish a questionnaire regarding their unmet needs for current disease management platforms. The study was approved by the Independent Ethical Committee of Tongji Hospital (NO: TJ-IRB20210912).

The AR-related questions were the following: (a) Did you have current or previous nose allergies? (b) Have you ever had a problem with sneezing or a runny or blocked nose in the past 12 months when you did not have a cold? (c) Have you ever been diagnosed with allergic rhinitis? (d) In the past 12 months, did you have itching, stuffy nose or sneezing when you touched grass, trees or flowers? (e) In the past 12 months, did you have itching, stuffy nose or sneezing when in contact with animals such as horses, dogs or cats with fur? (f) Have you ever had any current or previous symptoms of eye irritation (itchy, watery, redness)? (g) In the past 12 months, did you have a sandy feeling, redness, watery eyes or itchy eyes when you didn’t have a cold?

The asthma-related questions were the following: (a) Have you experienced any wheezing or whistling in the past 12 months? (b) Have you ever woken up at night because you felt chest tightness in the past 12 months? (c) Have you ever woken up at night with a cough in the past 12 months? (d) Have you had asthma attacks in the past 12 months? (e) Have you ever used aerosolized inhalers to help you breathe? (f) Have you ever used any medications (inhaled or oral) to reduce symptoms? (g) Have you experienced dyspnea or wheezing when you touched grass, trees or flowers? (h) Have you experienced dyspnea or wheezing when in contact with animals such as horses, dogs or cats with fur? (i) Have you ever had difficulty breathing or wheezing during or after exercise?

The physicians reviewed the history of suspicious respiratory allergic participants in a face-to-face manner and made diagnoses with reference to ARIA [20] and GINA [21] guidelines. In brief, those participants who fulfilled the following main criteria were regarded as AR: (a) symptoms of rhinorrhea, nasal obstruction or blockage, nasal itching, sneezing and postnasal drip that are reversible spontaneously or with treatment; (b) infections, drugs or other physical or chemical causes of rhinitis had been ruled out. Those who fulfilled the following main criteria were regarded as asthma: (a) recurrent episodes of wheeze, dyspnea, chest tightness or cough; (b) symptoms that occurred variably over time and varied in intensity. (c) symptoms that often occurred or were worse at night or on waking. (d) symptoms that were often triggered by exercise, laughter, allergens or cold air. (e) symptoms that often occurred with or worsened with viral infections.

### 2.2. Demographic Information

The demographic data on the students with respiratory allergies included age, gender, family history of allergic diseases, history of atopic dermatitis (AD), history of food allergy (FA) and the onset time of their symptoms. Their treatment status and satisfaction with treatment were also recorded. Satisfaction was scored from 0 to 10. A higher score represented more positive attitudes toward treatment.

### 2.3. The Brief Illness Perception Questionnaire (B-IPQ)

The B-IPQ was used to evaluate the parents’ perceptions of respiratory allergies. It is a universal questionnaire that had been validated as having acceptable psychometric properties [22]. The questionnaire consisted of 9 items, in which items 1 to 8 were multiple choice and item 9 was an open-ended question regarding the causes of their illness. The 8 clicker questions were (1) how much does your illness affect your life, (2) how long do you think your illness will continue, (3) how much control do you feel they have over your illness, (4) how much do you think their treatment can help your illness, (5) how much do you experience symptoms from your illness, (6) how concerned are you about your illness, (7) how well do you feel that you understand their illness, (8) how much does your illness affect you emotionally (e.g., make you angry, scared, upset or depressed). Each item was scored from 0 to 10. Higher scores represented more negative attitude to illness, except items 3, 4 and 7 were reversed. B-IPQ scores were calculated by adding up the scores for each question, with 3, 4 and 7 scored in reverse, and the total score ranged from 0 to 80 with higher scores reflecting more threatening illness perceptions.

### 2.4. The Asthma Knowledge Questionnaire (AKQ)

The AKQ was used to test the knowledge of the parents whose children had respiratory allergies. The items of the AKQ used in the study were adapted from a previously published questionnaire [23] and modifications were made by a multidisciplinary expert panel to ensure that the questionnaire was suitable for adoption in China. Six experts (three allergists, two respiratory experts and one nurse) were asked to discuss each item on the AKQ. Every question was scored by the six experts from 1 to 4 for content validity (1 = not relevant, 2 = somewhat relevant, 3 = quite relevant, 4 = highly relevant). The average score of each question was calculated. The modified AKQ consisted of 12 questions with the average scores ranked from 1st to 12th. The Cronbach’s alpha of the modified AKQ was calculated as 0.754, which revealed that it had good internal consistency. Consequently, the modified AKQ contained 12 true or false questions. The parents received one point for a correct answer and zero for a wrong answer for each question.

### 2.5. Allergy Platform Questionnaire (APQ)

The APQ was a questionnaire we designed to investigate the parents’ attitudes, unmet needs and expectations for current allergy platforms. It contained eight items, in which three items concerned the participant’s attitude and practice with current platforms; one item asked about the unmet needs as an open-ended question and four items dealt with the parents’ expectations for current platforms including preferred online platform, use pattern, frequency of updates and overall improvement. The items on unmet needs and expectations for overall improvement were multiple choice (up to five), and the other six items were single choice.

### 2.6. Statistical Analyses

Data were analyzed using SPSS 21.0 software. Descriptive parameters such as means and standard deviations were calculated for normally distributed continuous data, and frequencies and percentages were calculated for categorical data, where appropriate, 95% confidence intervals (95% CIs). Pearson’s χ^2^ test and Fisher’s exact test were used to determine the correlations between the categorical variables. The two-sample *t* test and one-way ANOVA were used to evaluate the continuous variables. The comparisons among groups were performed with LSD test or Tamhane’s T2 test. Multivariable linear regression was used to determine relationships between demographic characteristics and B-IPQ scores, the normality of continuous variables was assessed with the Shapiro–Wilk test. Binary logistic regression was used to explore the relationships between hospital treatment status and demographic variables and the B-IPQ scores. Factors through one-way binary logistic regressions with *p* < 0.2 were included in the multi-factor binary logistic analysis. Odds ratios (OR) and 95%CIs for potential factors were calculated. All tests were performed 2-tailed, and *p* < 0.05 was considered statistically significant.

## 3. Results

Of the 2430 distributed questionnaires, 2148 were returned (response rate: 88.4%). A group of 185 children were excluded due to incomplete questionnaires. Finally, a total of 1963 participants were included in the study; 930 (47.4%) were female. The average age was 10.6 ± 3.0 years old (ranging from 3 to 16 years old). According to their symptoms and doctors’ assessment, 576 (29.3%,95% CI 27.3–31.4%) children had respiratory allergies, of whom 504 (25.7%, 95% CI 23.7–27.6%) had been diagnosed with AR, 35 (1.8%, 95% CI 1.2–2.4%) with asthma, and 37 (1.9%, 95% CI 1.3–2.5%) with AR-complicated asthma (AR&Asthma). The average age of the respiratory allergies was 10.7 ± 3.0 years (ranging from 3 to 16 years old). Among the 576 respiratory allergy participants, 262 (45.5%) had family history of allergies, 137 (23.8%) had history of AD and 42 (7.3%) had a history of FA (Table 1).

The total B-IPQ score was 40.2 ± 10.9 in the respiratory allergies group. No differences were found between the AR, asthma and AR&Asthma groups (*p* > 0.05). However, the scores for items 1, 3 and 5 were different (*p* < 0.05) (Table 1). The history of AD and symptom onset within 12 months were correlated with high B-IPQ score (Table 2).

Only 269 (46.7%) respiratory allergy participants had received allergen tests: 109 (18.9%) of them had never received any treatment, and 95 (16.5%) had only received self-medication at home (Table 1).

**Figure 1:**
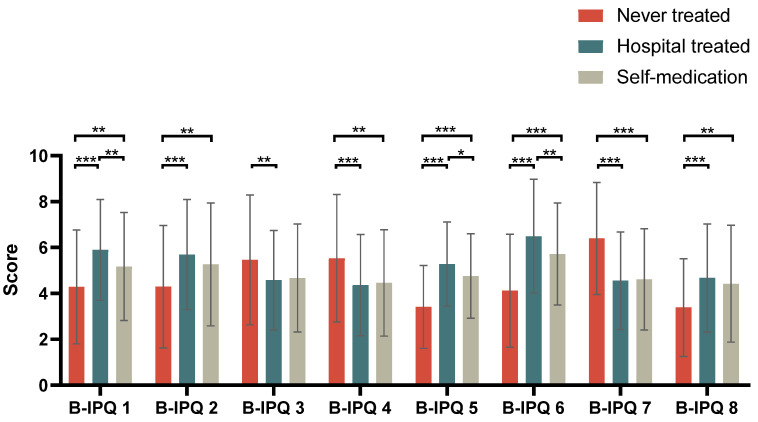
Comparison of B-IPQ item scores among participants with respiratory allergies in different health-seeking-behavior groups. Abbreviation: B-IPQ, Brief Illness Perception Questionnaire. * *p* < 0.05, ** *p* < 0.01, *** *p* < 0.001 (item 3, 4, 7 modified).

For the factors impacting the participants’ health-seeking behavior, univariate logistic regression analysis showed that participants with history of AD and those with higher B-IPQ scores were more likely to go to hospital for professional treatment. However, the parents of the participants with asthma did not have a strong desire to seek a doctor’s help. In the multivariate logistic regression analysis, a high B-IPQ score was an important factor in driving the participants to hospital; other demographic features such as age, gender, family history and complicated with AD/FA were not correlated with the health-seeking behaviors (Table 3). The total B-IPQ scores for no treatment, hospital-based treatment and self-medication were 36.9 ± 10.7, 41.5 ± 10.9 and 39.0 ± 10.4, respectively (*p* < 0.001). In general, the parents of the participants who never sought treatments had lower B-IPQ scores on most items compared with those who received treatments either by self-medication or in the hospital (Figure 1).

Among the 504 AR participants, 413 (81.9%) received treatment, among whom 329 (79.7%) received it in hospital; 172 (52.3%) were prescribed nasal spray and 222 (67.5%) were prescribed oral antihistamines. The other 84 (20.3%) were self-medicating. For the 35 asthma participants, 21 (60.0%) received treatment, 17 (81.0%) in hospital; another 8 (47.1%) had been prescribed inhalant corticosteroids, and the other 4 (19.0%) were self-medicating. For the 37 AR&Asthma participants, 33 (89.2%) had received treatment, 26 (78.8%) in hospital; 14 (53.8%) had been prescribed inhalant corticosteroids, and the other 7 (21.2%) were self-medicating. There were no differences in the parents’ satisfaction scores (ranging from 0 to 10, 0 for unsatisfied, 10 for very satisfied) to treatments between the self-medication and in-hospital treatment groups for AR, asthma and AR&Asthma (Table 1).

All the parents of the 576 participants with respiratory allergies had finished the AKQ. The participants were divided into an asthma group (*n* = 504) and a non-asthma group (*n* = 72). There was no significant difference in the total AKQ score between the two groups (*p* > 0.05). However, the correct response rate in the non-asthma group was higher than that in the asthma group for questions 2, 5, 6 and 7 in the AKQ (all *p* < 0.05) (Table 4).

**Figure 2:**
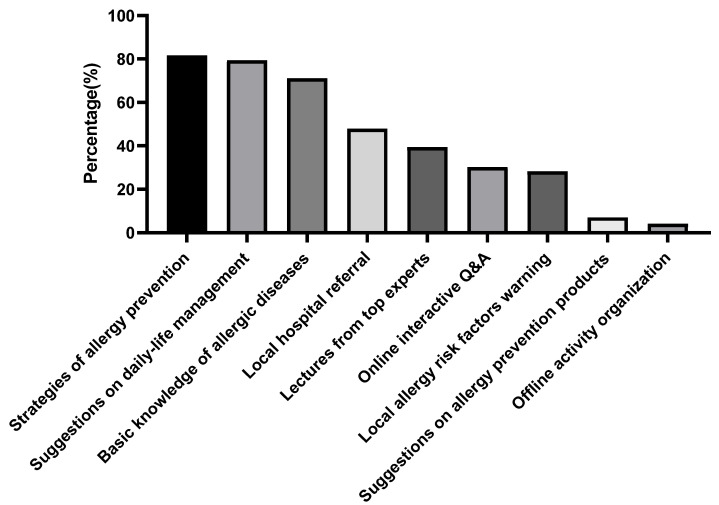
The online platform improvements the patients needed for obtaining professional information and recommendations. (*n* = 315).

Among the 576 parents of the participants with respiratory allergies, 568 (98.6%) had tried to obtain disease-management information from online platforms. For the online platforms, 64.1% of the parents preferred Wechat allergy public accounts, followed by Apps (17.1%), Wechat applets (12.4%) and websites (6.7%). Their user patterns included proactively browsing for interested content (49.2%), browsing content in spare time (36.5%) and passively browsing updated content (14.3%). The majority of the parents (76.8%) hoped to receive the updated news weekly, followed by 3 times weekly (15.9%) and daily (7.3%). However, 55.5% (315/568) were dissatisfied with current platforms. The reasons included incomprehensive contents on the illness (45.7%), lack of voice from leading experts (40.3%), too many advertisements (37.5%) and similar contents from different platforms (36.8%) (Table 5). The most desired areas of improvement for the platforms included content on preventing allergic diseases (81.6%), recommendations for allergic children in daily life (79.4%) and basic knowledge of allergy diseases (71.1%) (Figure 2).

## 4. Discussion

In our study, we found prevalence of respiratory allergies up to 29.3% among the 3- to 16-year-old population in Wuhan, which presented a great challenge to the society. However, the parents’ perceptions of these diseases were rather limited, and nearly 1/5 of the patients never received any treatment. With the development of social media, the parents tended to seek help or disease-related information from different online platforms. They had great expectations for these platforms. Considering that public education on respiratory allergies benefits patients as well as the society in the long run, we believe many of patients’ unmet needs should be addressed and improvements should be made accordingly for the current respiratory allergies management regimen.

Efforts to address these needs will be especially worthwhile when we consider the possible threat of these problems to the health care system in Wuhan, home to more than 13 million inhabitants. In many developed countries and regions, the prevalence of allergic diseases has slowed after a stage of rapid increase. For example, a study based on the ISAAC questionnaire showed that in Switzerland, the incidence of asthma and AR did not increase between 1992 and 2000 [24]. Studies found that the global incidence rate of asthma decreased with age, especially in those younger than age 10, in 1990 and 2017 [25]. However, in our study, the overall prevalence of AR was 27.6%, and for asthma, it was 3.7% in children ranging from 3 to 16 years old in Wuhan, similar to studies in Central China. These findings were not only very close to what was reported in another study conducted in 2018 [26], which showed prevalence of AR of 28.6% in 6- to 12-year-old students in urban area of Wuhan, but also to another epidemiological study conducted in 2009 [10]. The latter showed that the self-reported AR was 27.1% in 3- to 16-year-old children in Wuhan. These studies suggested that the prevalence of AR in this area might have reached a peak stage. Our speculation was also supported by the data from a nationwide epidemiological study that showed the prevalence of self-reported AR in Wuhan was 16.2% in 2005 and 17.2% in 2011 [1]. To date, the epidemiological studies of asthma in Wuhan have been limited. One study found that the self-reported lifetime adult asthma in Wuhan was 3.17% [27], which was slightly lower than that in our study. A cross-sectional study conducted in two periods (1993–1996, 2017–2018) spanning more than twenty years also showed a downward trend in adult asthma in Wuhan [28]. Although people may happy to see the mitigation of respiratory allergies, it is still a significant threat to health care when we take the large population in this area into account.

What aggravates the threat is that the patients’ perceptions of these diseases were very limited despite the numerous patients who were affected by respiratory allergies. In our study, we used the B-IPQ to evaluate the parents’ perceptions of respiratory allergies. B-IPQ is a nine-item scale designed to rapidly assess the cognitive and emotional representations of illness and has been widely used in many studies including AR-related [29,30,31,32] studies. In our study, we found the B-IPQ score was 40.2 ± 10.9 in respiratory allergies, which was even lower than that in Type 1 diabetes [29,33], suggesting that overlooking respiratory allergies was very common among the patients and their parents. It’s easy to understand that the perception of diseases has strong correlation with healthcare-seeking behaviors. Our study found that high B-IPQ score was a key factor driving the patient to go to hospital for professional help. Under the context of low B-IPQ scores among the studied population, it’s not strange that around one fifth of our patients never received any treatment and less than half of the patients had undergone further diagnostic procedure such as allergen tests. We also found the history of AD was a factor correlating with B-IPQ score, which might drive them to hospital. However, those patients with asthma were less likely to go to hospital. In general, the parents’ perceptions of respiratory allergies are far from adequate in the 3- to 16-year-old population.

Along with the low B-IPQ scores, the respiratory allergy patients’ knowledge of asthma was also poor. Asthma is a relatively severe condition of respiratory allergies and sometimes can be fatal. AR had been identified to be a crucial risk factor of asthma in many studies [34,35]. There was also evidence suggesting that early intervention of AR such as allergen immunotherapy precluded the development of asthma [36,37,38,39]. Raised awareness of asthma will push AR patients to take more actions to avoid developing asthma. Our study found overall accuracy on the AKQ among the respiratory allergy patients of around 75% and no differences were found between the AR and asthma groups, which indicated that their knowledge of asthma was insufficient. To our surprise, we found higher accuracy among the AR patients on some items than in the asthma patients. Considering that the items reflected important information about the management of asthma in daily life, it is imperative to promote health education in respiratory allergy patients especially in asthmatics.

The urgency of the matter is further supported by the patients’ dissatisfaction with the channels available to them. In contrast to face-to-face education, our study indicated that the majority of respiratory allergy patients preferred to seek medical information online. The online platforms provided massive information regarding the prevention and treatment of respiratory allergies in a convenient manner. However, more than half of our parents were dissatisfied with current platforms even though the information was easy to access, which implied a huge gap between the online disease content providers and the needs of the patients. In our study, the most common reasons for their dissatisfaction were that the contents were not so reliable and sometimes too commercial.

The patients’ expectations revealed in our study also necessitate a role health workers can play. We found that the main demands from the parents were more professional and comprehensive contents that could cover the major aspects of disease managements. Ideally the contents would be provided by authoritative physicians in allergies. In a sense, professional health care workers, especially the top experts in this field, may need to pay more attention to the popular online information on respiratory allergies so that more patients would be influenced by and benefit from it. In addition, the parents had very low willingness to participate in offline education activities, which means the patterns of physician–patient interactions should be adjusted accordingly in further medical practice. In fact, the online management of AR and other allergic diseases [40,41] was proposed and applied in some European countries in recent years, and it proved to be very successful.

We also realize the limitations of our study. Firstly, the diagnosis of respiratory allergies was based on symptoms, and no further examinations (such as allergen tests, lung function tests, etc.) were performed, which might have resulted in an overestimation of the prevalence of respiratory allergies in the population, as is often the case with questionnaire-based studies. Although we tried to minimize the bias by conducting a face-to-face interview with the participants with suspicious respiratory allergy symptoms and making diagnoses according to current guidelines, we still need to emphasize that the data on the prevalence of respiratory allergies obtained in our study and the subsequent scores on the B-IPQ and other questionnaires should be interpreted with caution. Secondly, all the questionnaires were answered by the parents, which could not reflect what the patients thought, especially for adolescents. Thirdly, the population in our study was not very large, and the overall questionnaire response rate was 80.7% (1963/2430), which might have led to bias when the prevalence of respiratory allergies was estimated. Finally, the COVID-19 pandemic might have had impacts on general medical care. For example, people might have limited going to hospital and worn facemasks in their daily lives. The changes in people’s lifestyles could have impacted our conclusions, although during the study period (June 2021 to July 2021), the COVID-19 pandemic had been well controlled in our area, and only one COVID-19 case was identified.

In conclusion, we find that the prevalence of respiratory allergies in the 3- to 16-year-old population in Wuhan is high, which is similar to other studies. However, the parents’ perceptions of respiratory allergies and knowledge of asthma are insufficient. To better manage these diseases, it is crucial to increase parents’ awareness of the illness and facilitate their access to informative and professional platforms. Allergists are also urged to deliver professional contents online. The improvement of online platforms should be given attention in future clinical practice.

## Figures and Tables

**Table 1:** Descriptive characteristics of the patients with respiratory allergies (*n* = 576).

	Respiratory Allergies			F/χ^2^	*p* Value
	AR	Asthma	AR&Asthma		
	(*n* = 504)	(*n* = 35)	(*n* = 37)		
Age, Years	10.8 ± 2.9	10.4 ± 2.9	9.0 ± 4.3	3.418	0.04 *
Gender				2.860	0.239
Male	293 (58.1)	18 (51.4)	26 (70.3)		
Female	211 (41.9)	17 (48.6)	11 (29.7)		
Family history				0.324	0.850
No	277 (55.0)	18 (51.4)	19 (51.4)		
Yes	227 (45.0)	17 (48.6)	18 (48.6)		
History of AD	118 (23.4)	9 (25.7)	10 (27.0)	0.325	0.850
History of FA	33 (6.5)	4 (11.4)	5 (13.5)	3.844	0.143
Symptom onset				43.960	0.000 *
<12 months	381 (75.6)	9 (25.7)	21 (56.8)		
>12 months	123 (24.4)	26 (74.3)	16 (43.2)		
B-IPQ score	40.3 ± 10.8	37.8 ± 13.7	41.6 ± 10.0	1.179	0.308
B-IPQ1	5.4 ± 2.3	5.8 ± 2.9	6.4 ± 2.2	3.66	0.026 *
B-IPQ2	5.4 ± 2.5	4.7 ± 2.8	5.2 ± 2.4	1.511	0.222
B-IPQ3	4.9 ± 2.3	3.7 ± 2.8	4.5 ± 2.5	4.310	0.014 *
B-IPQ4	4.6 ± 2.3	4.6 ± 3.1	4.4 ± 2.4	0.126	0.882
B-IPQ5	4.9 ± 1.9	3.9 ± 2.3	5.5 ± 1.8	6.034	0.003 *
B-IPQ6	5.9 ± 2.6	5.7 ± 3.0	6.2 ± 2.5	0.357	0.700
B-IPQ7	4.9 ± 2.3	5.2 ± 2.9	4.6 ± 2.4	0.392	0.678
B-IPQ8	4.4 ± 2.3	4.2 ± 2.9	4.7 ± 2.6	0.400	0.671
Treatment status				12.001	0.017 *
Never	91 (18.1)	14 (40.0)	4 (10.8)		
Hospital-based treatment	329 (65.3)	17 (48.6)	26 (70.3)		
Satisfaction	6.7 ± 2.1	7.8 ± 2.8	6.8 ± 2.0	1.963	0.142
Self-medication	84 (16.7)	4 (11.4)	7 (18.9)		
Satisfaction	7.2 ± 1.9	8.0 ± 2.4	7.9 ± 1.3	0.715	0.492
Receiving allergen test				5.703	0.058
No	268 (53.2)	24 (68.6)	15 (40.5)		
Yes	236 (46.8)	11 (31.4)	22 (59.5)		

Abbreviations: AD, atopic dermatitis; FA, food allergy; AR, allergic rhinitis; B-IPQ, Brief Illness Perception Questionnaire. * *p* < 0.05.

**Table 2:** Multiple linear regression model of B-IPQ scores in the respiratory allergy group.

	B-IPQ		
	Coefficients	95%CI	*p* Value
(Constant)	38.379	33.982, 42.775	0.000
Age	0.145	−0.156, 0.445	0.345
Gender	0.800	−0.964, 2.564	0.373
Family history	−0.738	−2.494, 1.019	0.410
History of AD	3.437	1.358, 5.516	0.001 *
History of FA	0.799	−2.626, 4.224	0.647
Symptom Onset	−5.284	−7.310, −3.258	0.000 *
Asthma	0.013	−3.772, 3.797	0.995
AR&Asthma	2.452	−1.166, 6.071	0.184

Abbreviations: AD, atopic dermatitis; FA, food allergy; AR, allergic rhinitis; B-IPQ, Brief Illness Perception Questionnaire. * *p* < 0.05.

**Table 3:** Factors associated with health-seeking behavior (going to hospital) in participants with respiratory allergies.

	Univariate Binary Logistic Regression		Multivariate Binary Logistic Regression	
	OR (95%CI)	*p* Value	OR (95%CI)	*p* Value
Age	0.971 (0.917, 1.029)	0.319	-	-
Gender	0.820 (0.581, 1.159)	0.261	-	-
Family history	1.158 (0.821, 1.634)	0.402	-	-
Diagnosis			-	-
AR		0.110	-	0.151
Asthma	0.502 (0.253, 0.999)	0.050	0.508 (0.250, 1.033)	0.062
AR&Asthma	1.257 (0.607, 2.605)	0.538	1.169 (0.556, 2.458)	0.680
Symptom onset	1.055 (0.722, 1.541)	0.782	-	-
History of AD	1.659 (1.086, 2.534)	0.019	1.431 (0.923, 2.217)	0.109
History of FA	2.109 (0.989, 4.500)	0.054	1.917 (0.884, 4.161)	0.100
B-IPQ score	1.032 (1.015, 1.048)	0.000	1.028 (1.011, 1.045)	0.001 *
B-IPQ1	1.254 (1.160, 1.355)	0.000		
B-IPQ2	1.162 (1.084, 1.246)	0.000		
B-IPQ3	0.912 (0.848, 0.980)	0.012		
B-IPQ4	0.889 (0.827, 0.955)	0.001		
B-IPQ5	1.429 (1.293, 1.580)	0.000		
B-IPQ6	1.295 (1.204, 1.394)	0.000		
B-IPQ7	0.824 (0.763, 0.890)	0.000		
B-IPQ8	1.158 (1.074, 1.247)	0.000		

Abbreviations: AD, atopic dermatitis; FA, food allergy; AR, allergic rhinitis; B-IPQ, Brief Illness Perception Questionnaire; OR, odds ratio; CI, confidence interval. * *p* < 0.05.

**Table 4:** Comparison of correct response rates for the Asthma Knowledge Questionnaire items between the asthma and non-asthma groups.

Items	Correct Response	Percent Correct (Non-Asthma, *n* = 504)	Percent Correct (Asthma, *n* = 72)	χ^2^/t	*p* Value
1. Coughing is not a symptom of asthma	F	279 (55.4)	46 (63.9)	1.865	0.172
2. Smoking in the home can make a child’s asthma worse.	T	488 (96.8)	65 (90.3)		0.017 *
3. If asthma symptoms such as tightness and wheezing do not occur for several years, a child has outgrown his/her asthma.	F	300 (59.5)	37 (51.4)	1.717	0.190
4. Asthma is an emotional or psychological disease.	F	374 (74.2)	54 (75.0)	0.021	0.885
5. Anger, crying or laughing can start an asthma attack	T	425 (84.3)	49 (68.1)	11.444	0.001 *
6. If you don’t have asthma by the time you are 40 years old, you will never get it.	F	454 (90.1)	59 (81.9)	4.280	0.039 *
7. Children with asthma should not play sports for which they have to run a lot.	F	384 (76.2)	43 (59.7)	8.910	0.003 *
8. An allergen is the antibody missing in people with asthma.	F	222 (44.0)	34 (47.2)	0.257	0.612
9. It is possible for your asthma to be worse without noticing a change in your breathing.	T	488 (96.8)	69 (95.8)		0.720
10. Exercising in cold weather can start an asthma attack.	T	413 (81.9)	55 (76.4)	1.276	0.259
11. Fish and birds are both good pets for a child with asthma.	F	398 (79.0)	57 (79.2)	0.001	0.969
12. Fewer people have asthma today than 10 years ago.	F	387 (76.8)	58 (80.6)	0.510	0.475
Total score		9.2 ± 2.5	8.7 ± 2.8	1.450	0.147

* *p* < 0.05.

**Table 5:** Dissatisfaction with and necessary improvements to the online platform in the three groups (*n* = 315).

	Total Patients (*n* = 315)	AR (*n* = 273)	Asthma (*n* = 15)	AR&Asthma (*n* = 27)	χ^2^	*p* Value
Dissatisfaction	315 (54.7)	273 (54.2)	15 (42.9)	27 (73.0)	7.024	0.03 *
1. The staff is not professional, and there is no explanation from the top allergy medical experts in China;						
	127 (40.3)	102 (37.4)	6 (40.0)	19 (70.4)	11.126	0.004 *
2. The content is not professional enough, and much of the content is too simple and similar to other platforms;						
	116 (36.8)	99 (36.3)	5 (33.3)	12 (44.4)	0.789	0.674
3. The content is too academic for the general public to understand;						
	109 (34.6)	91 (33.3)	4 (26.7)	14 (51.9)	4.162	0.125
4. The content is not comprehensive in initial medical guidance, diagnosis, treatment, prevention, life-style management and other aspects;						
	144 (45.7)	125 (45.8)	5 (33.3)	14 (51.9)	1.337	0.512
5. The update is not timely enough, and the interval between releasing new content is too long;						
	75 (23.8)	64 (23.4)	5 (33.3)	6 (22.2)	0.808	0.668
6. Push too frequently, too many updates are pushed daily, but the quality is not high, affecting the user experience;						
	63 (20.0)	57 (20.9)	2 (13.3)	4 (14.8)	1.002	0.606
7. Lack of timely interaction, online consultation function;						
	56 (17.8)	52 (19.0)	0 (0)	4 (14.8)	3.664	0.152
8. The information is too general, lacking local characteristics and detailed guidance in daily life;						
	55 (17.5)	48 (17.6)	1 (6.7)	6 (22.2)	1.457	0.496
9. The content is too commercial, with too many advertisements and product links						
	118 (37.5)	105 (38.5)	4 (26.7)	9 (33.3)	1.059	0.589

Abbreviation: AR, allergic rhinitis. * *p* < 0.05.

## Data Availability

Not applicable.
